# Computational Drug Design Applied to the Study of Metabotropic Glutamate Receptors

**DOI:** 10.3390/molecules24061098

**Published:** 2019-03-20

**Authors:** Claudia Llinas del Torrent, Laura Pérez-Benito, Gary Tresadern

**Affiliations:** 1 Laboratori de Medicina Computacional Unitat de Bioestadistica, Facultat de Medicina, Universitat Autónoma de Barcelona, 08193 Bellaterra, Spain; claudiallinasdeltorrent@gmail.com; 2 Computational Chemistry, Janssen Research & Development, Janssen Pharmaceutica N. V., Turnhoutseweg 30, B-2340 Beerse, Belgium; lperezb1@its.jnj.com

**Keywords:** mGlu, mGluR, homology model, virtual screening, molecular dynamics, allosteric modulator, GPCR, ligand-based design, structure-based design

## Abstract

Metabotropic glutamate (mGlu) receptors are a family of eight GPCRs that are attractive drug discovery targets to modulate glutamate action and response. Here we review the application of computational methods to the study of this family of receptors. X-ray structures of the extracellular and 7-transmembrane domains have played an important role to enable structure-based modeling approaches, whilst we also discuss the successful application of ligand-based methods. We summarize the literature and highlight the areas where modeling and experiment have delivered important understanding for mGlu receptor drug discovery. Finally, we offer suggestions of future areas of opportunity for computational work.

## 1. Introduction

**Figure 1:**
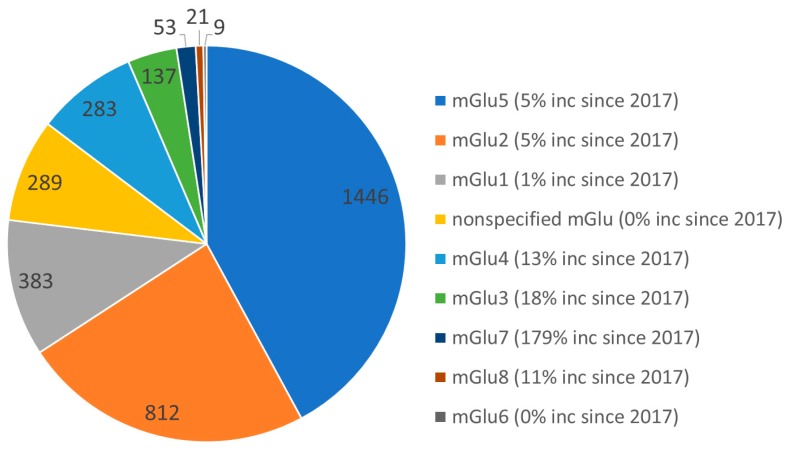
Pie chart showing reported ligands for mGlu receptors in the Thomson Reuters Integrity database. The most explored are mGlu_5_, mGlu_2_, and mGlu_1_. Extracted on 29 January 2019 and compared with results from March 2017.

Glutamate is the major excitatory neurotransmitter in the central nervous system (CNS) and modulates synaptic responses by activating ionotropic glutamate (iGlu) receptors and G protein-coupled receptors (GPCRs) termed metabotropic glutamate (mGlu) receptors. GPCRs are the largest class of membrane proteins in the human genome and are crucial for cell signaling [1]. The metabotropic glutamate (mGlu) receptors are family C GPCRs that participate in the modulation of synaptic transmission and neuronal excitability throughout the CNS. To date, eight mGlu receptor subtypes and multiple splice variants have been cloned and classified into three groups based upon sequence homology, pharmacological profile and preferential signal transduction pathways [2]: Group I mGlu receptors (mGlu_1_ and mGlu_5_), group II (mGlu_2_ and mGlu_3_) and group III receptors (mGlu_4_, mGlu_6_, mGlu_7_ and mGlu_8_). They have emerged as interesting drug targets [3] and pharmaceutical research has primarily focused on the pharmacology and development of ligands acting at groups I and II, whereas group III receptors have started to attract attention more recently (Figure 1) [4].

Group I receptors, mGlu_1_ and mGlu_5_, may be useful targets for psychiatric disorders via their indirect modulation of NMDA receptors [5]. They are coupled to protein kinase C (PKC) via G_q_ protein and are ubiquitously expressed on postsynaptic excitatory terminals in limbic brain regions that are involved in motivational, emotional and cognitive processes [6]. For instance, mGlu_5_ and NMDA receptors are co-expressed at GABAergic interneurons, and are not only connected via intracellular signaling pathways but also physically through scaffolding proteins [7,8]. These receptors may have the potential for therapeutic intervention of schizophrenia. Meanwhile, group II mGlu receptors reduce transmission at glutamatergic synapses in brain regions such as the prefrontal cortex and hippocampus where excessive glutamatergic transmission may be implicated in the pathophysiology of anxiety and schizophrenia [9]. It is therefore hypothesized that activation of group II mGlu receptors, mGlu_2_ and mGlu_3_, may provide anxiolytic and/or antipsychotic effects [5]. Finally, the group III receptors, mGlu_4_, mGlu_6_, mGlu_7_ and mGlu_8_, are much less explored but offer huge potential. Ligands of mGlu_4_ for example have recently shown to be neuroprotective and beneficial in animal models of Parkinson’s disease, pain, and anxiety [10]. Furthermore, ligands of mGlu_7_ and mGlu_8_ receptors have possible indications in neurodevelopmental disorders and anxiety, post-traumatic stress, Parkinson’s, and multiple sclerosis [11,12].

**Figure 2:**
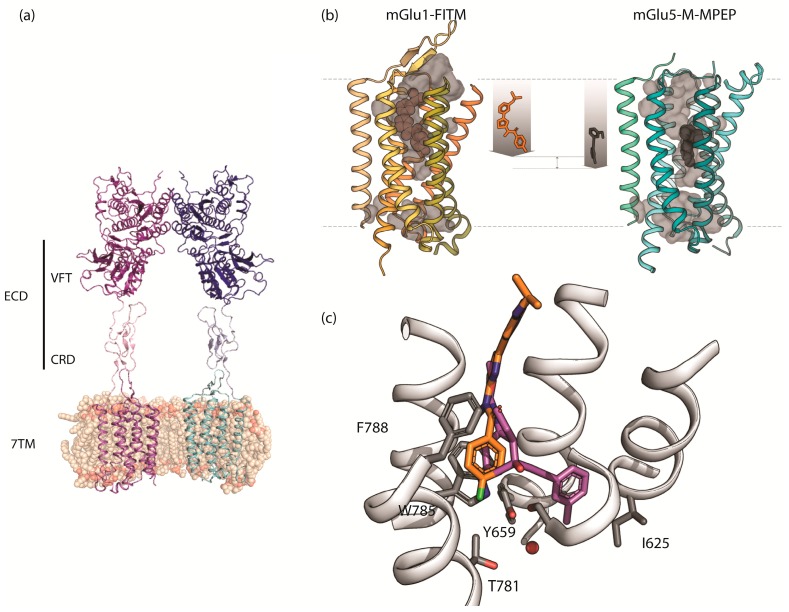
The structure of mGlu receptors: (**a**) Structure of the full length apo mGlu_5_ receptor homodimer showing the VFT, CRD, and 7TM domains (PDB 6N52); (**b**) X-ray structures of mGlu_1_ and mGlu_5_ receptor 7TMs showing the different depth of binding for NAMs. (**c**) Close up view of the X-ray structures of the 7TM domains showing the overlay of NAMs FITM (orange) and mavoglurant (magenta) bound at mGlu_1_ and mGlu_5_ receptors with several amino acids labelled.

The mGlu receptors form cysteine linked dimers. Their structures include a characteristic Venus fly-trap (VFT) extracellular agonist-binding domain, a 7-transmembrane (7TM) domain, and a cysteine rich domain (CRD) that connects the two (Figure 2) [13]. The first structures of a full length mGlu receptor were recently deposited in the protein databank (PDB id 6N51 and 6N52). Solved by electron microscopy they reveal how activation proceeds by compacting the intersubunit VFT dimer interface, bringing the CRDs into proximity, and in-turn enabling the 7TM domains to reposition closer together to initiate signaling [14]. The extracellular loop 2 of the 7TM makes crucial interactions with the CRD. This is a huge breakthrough for the understanding of mGlu receptor function. Future work will likely improve the resolution, and study local interactions and conformational changes, helping to understand the 7TM movements and interactions that occur with activation. Previously, the VFT and 7TM domains have been solved by X-ray crystallography. The crystal structures for the extracellular VFT domains of mGlu’s 1, 2, 3, 5, 7 and 8 reveal the closed agonist-bound active conformations as well as resting and inactive conformations [15]. Meanwhile, the crystal structures of the 7TM domain of mGlu_1_ and mGlu_5_ have shown the binding site of allosteric ligands in an analogous position to the orthosteric pocket of the class A receptors. The computational study of class C GPCRs has benefited greatly from the publication of the mGlu_1_ and mGlu_5_ 7TM crystal structures in 2014 [16,17]. Despite their similarity, the mGlu_5_ structure was crystallized with a NAM (mavoglurant) binding in a deeper pocket compared to the NAM (FITM) bound to mGlu_1_ (Figure 2). Two more recent studies provided more mGlu_5_ 7TM X-ray crystallographic information revealing subtle differences in binding modes for different NAMs [18,19].

As these structures suggest, mGlu receptor drug discovery has focused on targeting both orthosteric and allosteric ligands. Ligands targeting the orthosteric site are often amino-acid like, but reports have shown that group II mGlu receptor ligands can achieve brain penetration and selectivity versus other members of the mGlu family [20]. Despite this, the mGlu receptor family has seen much effort dedicated to allosteric drug discovery which provides ways to modulate GPCRs for potential therapeutic benefit [21]. Allosteric modulators of mGlu receptors avoid amino-acid (glutamate-like) chemistry that can be difficult for brain penetration. The allosteric site is less conserved, helping the identification of selective ligands. Furthermore, allosteric modulators typically only modify receptor response in the presence of the endogenous ligand thus avoiding receptor desensitization or other unwanted-effects of agonist action. Allosteric modulators are classified as either positive allosteric modulators (PAMs) or negative allosteric modulators (NAMs) that either enhance or inhibit the response to glutamate respectively. Silent allosteric modulators (SAMs) are ligands that occupy the allosteric pocket but do not modify activity.

Given the substantial interest in this target class, but the challenges for structure-based drug design, the mGlu receptor field has seen a variety of alternative computational approaches to aid drug discovery. Computationally assisted drug design can offer many varied techniques useful in lead discovery [22], virtual screening (VS) [23] and in lead optimization [24,25,26]. In this manuscript we review the reports of in silico methods applied to this GPCR subfamily. It is found that a wide range of methods have been used, from applying pharmacophores and other tools for VS, modeling and docking to understand binding modes, to molecular dynamics (MD) to understand origins of functional effect of allosteric modulation. Thus, this target family has spurred diverse and creative approaches that can be of interest for future drug discovery target families.

## 2. Group I mGlu Receptors

### 2.1. mGlu_1_ Receptor

The mGlu_1_ receptor is an attractive target for several psychiatric diseases and neurological disorders [27]. Research on its structure has also benefited the understanding of the features needed to selectively target both orthosteric and allosteric binding sites of this receptor. Many early mutagenesis studies using site-directed mutagenesis, truncations and chimeras of the receptor obtained details on mGlu_1_ functional behavior. Some papers described the role of C-terminal residues in protein coupling [28] or the ones involved in dimerization [29], but most efforts focused on elucidating the important residues for ligand recognition [30,31,32,33,34,35,36,37].

**Figure 3:**
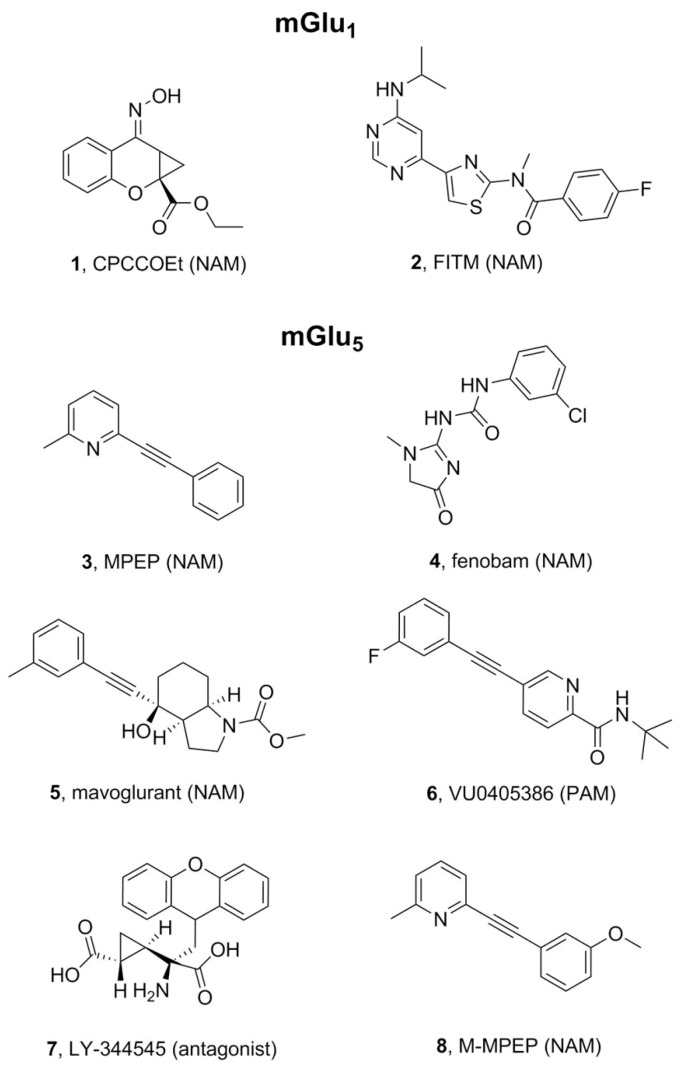
Chemical structures of selected ligands for mGlu_1_ and mGlu_5_ receptors.

In the late 1990s the first mGlu_1_ selective non-competitive antagonists were described, suggesting the existence of an alternative binding site for these compounds. Several mutagenesis studies began to propose mGlu_1_ residues involved in the recognition of these non-competitive antagonists, such as CPCCOEt (**1**, Figure 3), suggesting an overlapping binding site in the 7TM domain of the receptor [30,31]. In 2003, Malherbe et al. built a computational homology model of the receptor based on the available bovine rhodopsin crystal structure which allowed to specifically mutate binding pocket facing sidechains and pinpoint a cluster of aromatic residues in TM6 as important for mGlu_1_ NAM activity [32]. These residues coincided with the non-orthosteric antagonistic binding site noted in the previous mutagenesis studies.

In the absence of a solved crystal structure of the full receptor or 7TM, scientists sought other approaches to understand the compound selectivity of mGlu_1_ receptor antagonists such as the VS performed in 2006 by Noeske et al [38]. They used self-organizing map (SOM) clustering algorithms and a topological pharmacophore descriptor to predict the potential activities of some known NAMs. With this VS strategy, they were able to discriminate between antagonists of mGlu_1_ and mGlu_5_ useful for early recognition of potential selectivity concerns in lead discovery. In that same year, researchers performed a docking study using the binding site domain (orthosteric) crystal structure of the mGlu_1_ receptor. They generated homology models for ECDs of other class C receptors and observed that their VS approach was able to recognize the best ligands in most cases, with a combination of different docking and scoring functions being required for optimal results [39].

A similar homology model of mGlu_1_ was used by Merz Pharmaceuticals’ scientists in studies from 2007 and 2008. The first was a ligand-based VS study, in which they analyzed the binding mode of two representative mGlu_1_ NAM hits describing potential interaction patterns [38]. In Vanejevs et al. the model was used for the SAR interpretation of novel modulators [40]. They developed a pharmacophoric hypothesis for mGlu_1_ antagonists based on previously described ligands and used this to perform VS of a large compound database. Lead optimization on the hit scaffold was carried-out, and a small library of compounds was docked into the homology model to predict the binding mode and help SAR understanding.

In 2014, the release of the mGlu_1_ 7TM crystal structure bound to NAM FITM (**2**, Figure 3) [17] increased the scope of modeling opportunities. In 2015 Harpsøe et al. performed an extensive computational analysis of all the mGlu receptors, merging information on experimental mutagenesis with the new insights from the crystal structures to predict binding modes and discuss common interactions [41]. The work points out several residue hotspots and characteristic features within the allosteric binding pocket of the different mGlu receptors that can explain NAM subtype selectivity. This information has since been incorporated into the GPCR database [42]. With the new crystal structure information more reports began to emerge. For instance, Jiang et al. used pharmacophore models based on known mGlu_1_ NAM ligands to VS a database of Chinese herbs. The hit compounds were then docked into the mGlu_1_ crystal structure suggesting new possible allosteric ligands, although this was not confirmed experimentally [43].

A hierarchical VS approach was employed to identify new micromolar mGlu_1_ receptor NAMs [44]. A homology model of mGlu_1_ was built based on the D3 receptor and refined using MD simulations. Known mGlu_1_ antagonists were then used to build a pharmacophore model, as well as a recursive partitioning model, a Bayesian model, and a support vector machine (SVM) model. VS was initially performed using a pharmacophore guided structure-based approach, then hits were classified as likely mGlu_1_ actives with the remaining models. After applying this approach to commercially available compounds new active mGlu_1_ allosteric modulators were identified. Retrospective docking validation of the homology model with the released mGlu_1_ crystal suggested the predicted binding mode of the hits obtained.

Also in 2015, the mGlu_1_ receptor 7TM crystal structure was used as a test case for studying the functional group affinity patterns for its occluded allosteric binding site [45]. The site identification by ligand competitive saturation (SILCS) methodology uses fragment sampling to map free energy affinity patterns of functional groups at protein surfaces. MD of mGlu_1_ in a membrane and with different solute fragments was performed and the affinity patterns were obtained based on probability analyses. The method revealed the largely hydrophobic nature of the mGlu_1_ allosteric binding site consistent with the lipophilic allosteric ligands [46].

Bai et al. combined three computational methods to study the mGlu_1_ receptor [47]. They performed MD simulations of the receptor wild type and T815M and T805A mutants. Weak interaction analysis and free energy calculations based on the outcome of the simulations provided insights into the dimeric packing and allosteric regulation mechanism of the receptor. Free energy calculations showed that residues T748, C746, K811 and S735 form the secondary binding pocket, where FITM binds before escaping the receptor, suggesting these amino acids are relevant for the dynamic binding of this NAM.

Recently in 2018, quantum mechanical methods were used to study the binding site of FITM in the mGlu_1_ 7TM domain and compare it with twelve close analogs covering a broad range of affinity [48]. The binding site geometries were optimized using the “our own N-layered integrated molecular orbital and molecular mechanics” (ONIOM) method along with the fragment molecular orbital method with energy decomposition analysis (FMO-EDA). The results showed that residues Q660^3.28^ and/or Y805^6.55^ were anchor points for all the FITM analogs, while the H-bond with T815^7.38^ determined only the orientation of very active molecules containing an amino substituent in the pyrimidine moiety (e.g., FITM). The orientation of the other parts of ligands resulted from hydrophobic interactions mainly with L757^5.44^, F801^6.51^, or W798^6.48^. The applied ONIOM/FMO–EDA approach facilitated the study of effects related to very small changes in the ligand structure and led to conclusions regarding the significance of individual interactions in the allosteric binding pocket of mGlu_1_.

### 2.2. mGlu_5_ Receptor

Due to its interest for depression [49], migraine [50], fragile X-syndrome [51] and many other CNS disorders, there has been much effort to understand the mechanism of action and ligand interactions of this receptor.

**Figure 4:**
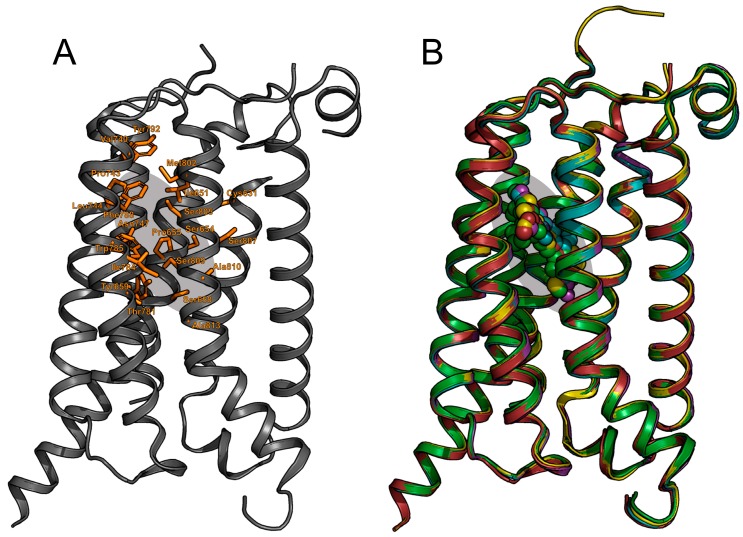
(**a**) The mGlu_5_ 7TM showing amino acids identified from experimental mutagenesis as important for allosteric modulator activity, compared with (**b**) the overlap of the subsequent X-ray crystal structures of multiple mGlu_5_ receptor NAMs.

Since the early 2000s, site-directed mutagenesis has been used frequently to characterize the allosteric binding pocket of the mGlu_5_ receptor (Figure 4) [52,53,54,55]. However, the mutagenesis data was best interpreted by performing homology modelling. First studies used bovine rhodopsin as the template since it was the only GPCR crystal structure available [32,52]. Later, class A 7TM GPCR structures were used instead, such as the β_2_ adrenergic receptor or the A_2A_ adenosine receptor [54,56]. These early homology models together with the mutagenesis data were used to hypothesize the binding modes of the mGlu_5_ ligands. Manual or computed docking of the ligands were carried out for ligand-protein interactions analysis [31]. In this sense, Gregory et al. wanted to understand the molecular determinants dictating allosteric modulator interactions in mGlu_5_ [57]. Site-directed mutagenesis was performed obtaining six main residues as key for ligand affinity. Many diverse allosteric ligands were docked into their homology model receptor based on β_2_ adrenergic receptor crystal. They observed a clear overlap in binding modes of the compounds confirming the location of the allosteric pocket in the 7TM domain of the receptor. Overall, the 7TM allosteric binding site defined by experimental mutagenesis and computational studies was later verified by the 7TM crystal structures (Figure 4).

Pharmacophore modeling was another computational tool used in the field to identify new allosteric modulators of both mGlu_1_ and mGlu_5_ receptors. Coupling of mutagenesis data with a 3D pharmacophoric model of the receptor was performed to elucidate the binding mode of MPEP (**3**, Figure 3) and fenobam (**4**, Figure 3) [58]. As mentioned in the mGlu_1_ section, scientists from Merz Pharmaceuticals built a pharmacophore model for mGlu_1_ allosteric modulators based on previously described ligands [40,59]. Given the high structural similarity between mGlu_1_ and mGlu_5_ allosteric pockets, this model was also used for the design of mGlu_5_ allosteric modulators. The pharmacophore model was used to screen a wide library of compounds and hit optimization led to highly potent mGlu_5_ NAMs and PAMs.

Given the difficulties to perform structure-based drug design for mGlu receptors it is understandable that many computational approaches instead relied upon ligand-based methods, especially machine learning methods for discovery of new compounds. One of the first uses of machine learning algorithms in this field was performed by Renner et al. in 2007. They used an artificial neural network (ANN) combined with a 3D pharmacophoric model for discovery of new NAMs of mGlu_5_. The VS resulted in the identification of three potent mGlu_5_-specific hits [60]. Later in 2010, a comparative molecular field analysis (CoMFA) 3D-QSAR approach was applied to a set of mGlu_5_ PAMs [61]. Mueller et al. [62] tried to overcome QSAR limitations by implementing artificial neural networks (ANN) approaches for the identification of new potential mGlu_5_ modulators. High throughput screening data were used to train an ANN model to predict mGlu_5_ NAM activity. The model was then applied to identify new NAM chemotypes by VS. The hits led to the identification of a series of allosteric modulators. Further work involved synthesizing MPEP analogues, then docking them into a homology model to understand their selectivity. An mGlu_5_ receptor model was used based on both β_2_ adrenergic receptor and A_2A_ receptor crystal structures, validated by previous mutagenesis studies. Docking the compound of interest into the model suggested its binding mode, indicating the relevance of certain residues such as N747, and S809 for NAM binding [63].

In 2014 Dalton et al. built an active state of the 7TM domain by homology modelling. Multiple templates such as β_2_ adrenergic receptor, sphingosine 1-phosphate receptor 1, dopamine D3 receptor and neurotensin receptor were used for the construction of both the inactive and active state models. Four NAMs and four PAMs previously described in other studies were docked into their respective receptor state model using an induced-fit method. PAM and NAM binding modes were compared and led to identification of amino acids S809, W785, T781 and Y659 as key residues [64].

The 7TM crystal structure of mGlu_5_ was published in 2014 and proved a turning point for mGlu_5_ targeted drug design. The previously obtained mutagenesis data and SAR studies could be mapped, (Figure 4). For instance, the above-mentioned study from Harpsøe et al. [41] merged large amounts of experimentally available mutagenesis data with a docking study of several mGlu NAM ligands. Despite many similarities between mGlu_1_ and mGlu_5_ structures, their allosteric pockets reveal the determinants of ligand selectivity. A key selectivity residue is the amino acid on TM3 at position 40: 3.40. In mGlu_1_, S668^3.40^ together with Thr815^7.33^ appear to induce selectivity for this receptor by H-bonding to the ligands. On the other hand, mGlu_5_ has a proline residue at the 3.40 position. The proline occupies less space compared to the bulkier side chains at this position in the other mGlu receptors allowing ligands to pass and bind deeper in mGlu_5_ (Figure 2). This hypothesis is supported by mutagenesis evidence in which mutations such as Pro655Phe, result in abolishment of NAM effects [54]. The comparison of the first crystal structures of mGlu_5_ [16] versus mGlu_1_ [17] confirmed this, all available mGlu receptor 7TM domain structures are summarized in Table 1. Later, Harpsøe et al. used the mGlu_5_ structure bound to mavoglurant (**5**, Figure 3) to place ligands in the binding site and establish that subtype selectivity is achieved by reaching the deep pocket of the receptor, and it can be further improved by interacting with S805 [41].

Along the same lines, a medicinal chemistry study by Anighoro et al. [65] used an homology model of mGlu_5_ based on the crystal structure of mGlu_1_. Docking of MPEP and fenobam (mGlu_5_ NAMs) was performed using an induced fit approach. MD simulations of these complexes were performed for refinement of the predicted binding modes, which were confirmed by previous mutagenesis data. The release of the mGlu_5_ receptor enabled definitive validation of the binding modes described in the work by docking the NAM compounds. They synthesized a new series of NAMs, docked them to the receptor structure and extracted the binding modes and SAR. The obtained insights on interaction modes of NAMs could be useful for the future design of high affinity mGlu_5_ modulators.

Feng et al. used the mGlu_1_ and mGlu_5_ crystal structures to model the whole family of 1–8 7TM domains and studied the binding modes of multiple allosteric ligands [66]. They compared the different interactions of PAMs and NAMs. They went on to construct a model of a full length mGlu_5_ receptor (amino acids 26 to 832). However, according to the report, it was only modeled in the monomeric form. This construct was submitted to a 100 ns MD simulation in the presence of an orthosteric antagonist-LY-344545 (**6**, Figure 3) and the mavoglurant NAM, as well as bound to the agonist glutamate and PAM-VU0405386 (**7**, Figure 3). They reported large conformational changes of glutamate bound within the orthosteric binding site as well as in the CRD when compared with the simulations performed using the antagonist + NAM. Within the 7TM they observed substantial differences in the behavior of the ionic-lock on the intracellular side of the receptor when comparing the antagonist+NAM with the agonist+PAM simulations. They also witnessed the intracellular opening of TM5 in the presence of PAM and suggested this may facilitate activation.

In 2017 the mGlu_5_ crystal structure was used to model MPEP activity versus mGlu_4_ and mGlu_5_ [67]. The results showed different interaction modes of this ligand with the receptors, which they argued can explain the opposite functionality of this molecule in the different contexts. Dalton et al. suggested two different ligand-receptor interactions given by H-bonding of MPEP with TM3 residues in mGlu_4_ or with TM7 residues in mGlu_5_. They also consider the depth of the allosteric pocket a key determinant for the binding modes difference, since 636^2.49^ residue in mGlu_4_ receptor is a less bulky G628^2.49^ in mGlu_5_ thus allowing the ligand to bind deeper into the allosteric pocket. The authors also proposed the occupancy of the binding pocket by a lipid when no modulator is present. Also in 2017, Bian et al. performed a purely in silico study to generate potential allosteric mGlu_5_ receptor ligands [68]. They fragmented a database of existing allosteric modulators and used these fragments to recombine and generate new ligands. Docking and scoring identified the top-ranking new molecules which were suggested to be potential new mGlu_5_ modulators. Later in 2018, Fu et al. [69] performed a computational study of the pharmacophoric binding of several mGlu_5_ NAMs identifying important amino acids for binding in the allosteric site. They used induced fit docking and MD approaches for their objective, and their results were supported by mutagenesis data.

In 2018, two more crystallographic structures of NAMs, M-MPEP (**8**, Figure 3) and fenobam, bound to the mGlu_5_ receptor 7TM domain were solved by Christopher et al [19]. Along with revealing the new structures they also performed a computational study of the role of water in the activity of mGlu_5_ NAMs. The crystal structures have already been used for some computational studies such as VS using a pharmacophoric model [70]. The pharmacophore was validated by induced-fit docking of the hit compounds, and ensuing MD simulations were performed for analysis of the specific ligand-receptor interactions. The same authors also performed a QSAR study of a thieno[2–b]pyridines series of mGlu_5_ receptor NAMs [71]. They first built an all-atom QSAR model from the pharmacophore hypothesis of this list of compounds. The model was used for induced-fit docking and binding free energy calculations, MD of the complex were run for stability verification. Again, the results of this paper are thought to contribute useful knowledge for future NAM drug design studies.

A recent study demonstrated for the first time the origin of the functional effect of NAMs and PAMs of the mGlu_5_ receptor, by studying close analogue molecular switches. A series of MD simulations elucidated the mechanisms by which minor changes in ligand scaffold can elicit completely opposite functional activity [72]. These molecular switches have only been described for mGlu_5_. An active-like model of the receptor was built using the mGlu_5_ 7TM crystal structure and the β_2_-Gs active-state complex (PDB ID 3SN6). The Gq protein was included in the model to obtain a fully active-like receptor state. By simulating NAMs bound to the inactive 7TM mGlu_5_ receptor crystal structures, and PAMs bound to the active-like model of mGlu_5_ three residues were identified to be crucial for the allosteric activation mechanism of the receptor: S658, Y659 and T781. The hypothesis is consistent with previously described residues in class A and class C receptors that are involved in the activation process, and their relevance is also supported by former mutagenesis studies. Additionally, water analysis of the systems enabled the identification of a key water molecule for stabilization of the inactive state of the receptor. The results can be extrapolated to additional mGlu_5_ NAM and PAM ligands, to understand how they induce their functional effect. This work contributes to better understanding of the mGlu_5_ allosterism and activation mechanisms and can be of great help for future drug design studies.

## 3. Group II mGlu Receptors

### 3.1. mGlu_2_ Receptor

Given its links to various neuropsychiatric disorders the mGlu_2_ receptor has been amongst the most studied from a medicinal chemistry point of view (Figure 1) [73,74,75,76,77,78]. Early computational modeling work involved generating mGlu_2_ receptor models and docking to support and explain site directed mutagenesis studies. This was particularly relevant to understand the mode of action and identify binding sites for allosteric modulators.

In 2009 and 2011 scientists from Roche (Basel, Switzerland) reported detailed mutagenesis studies of both the orthosteric and allosteric binding sites [79,80]. They modeled an antagonist binding mode in the closed inactive form of the mGlu_2_ VFT as well as the binding of two NAMs bound in the 7TM. The binding modes could explain the mutant observations but the 7TM modeling preceded the release of the mGlu_1_ and mGlu_5_ crystal structures. Instead, human β_2_ adrenoceptor was used as a structural template which may have led to the binding site being more extracellular facing than later revealed for mGlu receptors.

Subsequently, Janssen (Beerse, Belgium) scientists have also reported detailed experimental mutagenesis studies of mGlu_2_ PAMs and NAMs that were driven with computational modeling. In the period from 2015 to 2018 this group published several studies that confirmed an overlapping binding site and similar interactions amongst PAMs, and amongst NAMs, but with some interactions being unique to each set [81,82]. These extensive studies involved as many as 12 ligands and 39 mutants. Along with binding displacement studies, and deep analysis of structure activity relationships, this led to relatively confident predictions of the binding mode of the mGlu_2_ allosteric modulators. Furthermore, the binding hypothesis was confirmed by the computational targeted design of an mGlu_2_ allosteric covalent ligand [83].

**Figure 5:**
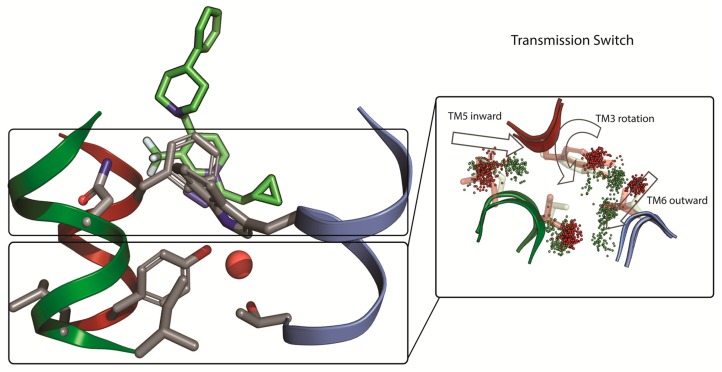
Origin of the functional effect of mGlu_2_ PAMs and mGlu_2_ NAMs. A combined experimental and computational study by Perez-Benito et al. [82] showed that PAMs and NAMs bind above the transmission switch but transmit a different conformational effect. Based on the modelling, amino acids in the transmission switch were proposed for experimental mutagenesis that confirmed their importance for receptor activation. Thus, validating a hypothesis of local allosteric modulator induced conformational changes that promote or block movements of amino acids responsible for functional activity, overall confirming the 7TM analogy with class A GPCRs.

Building upon this relatively solid understanding of the binding mode they went on to consider how the PAMs and NAMs achieve their functional effect. Classical MD simulations were performed for µs timescales using repeats and multiple ligands. It was consistently demonstrated that PAMs and NAMs induce different conformational behavior of amino acids in their direct vicinity (so-called trigger switch), that in turn modifies the conformational behavior of amino acids one turn below in the known transmission switch (Figure 5) [82,84,85]. The amino acids involved in the transmission switch correspond to the same functionally important positions known from class A GPCRs. Based on the modeling they went on to perform additional mutation of amino acids in the transmission switch and confirmed them to be crucial even for functional activity of glutamate alone. Thus, the hypothesis emerges whereby NAMs can block movements in this region, whereas PAMs promote sidechain movement, particularly of amino acids such as threonine 769 in transmembrane helix 6 (T769^6.44a.46c^) that can induce kinking in helices.

Another interesting modeling study used MD approaches to demonstrate cross talk between a heterodimer of mGlu_2_ and 5HT_2A_ receptors. Bruno et al. constructed the model and performed classical MD simulations that could explain the allosteric effect of mGlu_2_ receptor on 5HT_2A_ mediated response [86]. As this study predates the 7TM crystal structures of mGlu_1_ and mGlu_5_ receptors, the authors had to use the human β_2_ adrenergic receptor to model 5HT2A and bovine rhodopsin as a template for mGlu_2_. Nevertheless, the work suggested the TM4/TM5 interface was likely stable and preferred for initiating the allosteric effect between receptors. Another MD study investigated the conformational behavior of 7TM helix 8 in the mGlu_2_ receptor [87]. Little is known about the role of helix 8 in class C GPCRs. Extended MD simulations indicated that helix 8 adopts membrane-sensitive conformational states and is dependent on interaction with cholesterol, in short, the more cholesterol present the more stabilized the helix 8 structure.

In 2014 computational modeling of the mGlu_2_ receptor was used to assist an important study investigating the inter-monomeric rotation during activation of the mGlu_2_ receptor dimer [88]. Computational models of inactive and active dimeric complexes were built that were able to assist the definition of the cross-linking experiments and aid analysis. The modeling helped to reveal that TM4 and TM5 are mainly cross-linked in the inactive state, whilst TM6 constitutes the interface of the active dimer.

**Figure 6:**
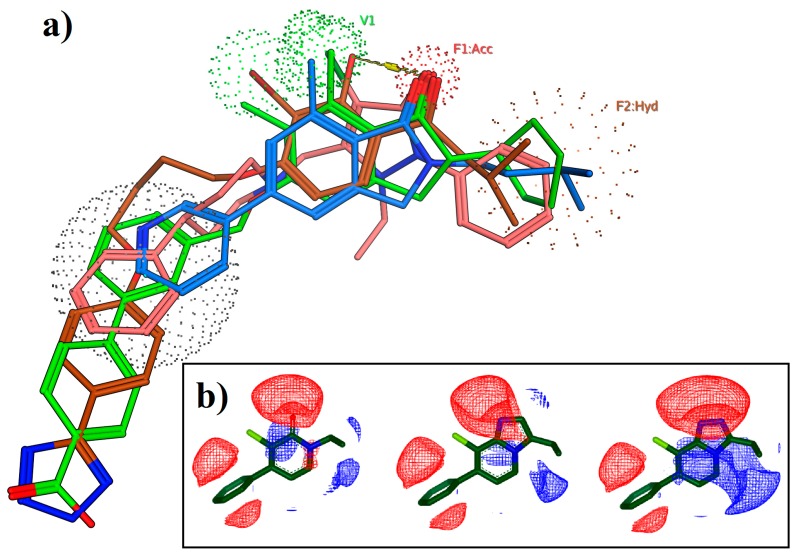
Ligand-based approaches leading to identification of new mGlu_2_ PAMs. (**a**) The structural alignment and pharmacophore of selected mGlu_2_ receptor PAMs. (**b**) The electrostatic fields showing the similarity between pyridone and subsequent imidazopyridine and triazopyridine scaffolds.

In 2010 scientists from Janssen applied computational ligand-based approaches to help identify new mGlu_2_ PAMs [89]. They showed how the ligands of several different mGlu_2_ PAM chemical series shared a common alignment and features (Figure 6). A database of virtual fragments was generated and searched using shape and electrostatic similarity methods to identify alternative scaffolds that mimic the steric hindrance and features but with alternative underlying chemical connectivity. This scaffold-hopping approach led to modification of a pyridone series to a novel imidazopyridine scaffold. Rapid decoration of the new scaffold resulted in ~100 nM mGlu_2_ PAMs amongst the first synthesized examples and subsequent optimization led to further potent advanced leads [75,84,85,90,91]. This application shows the power of relatively simple, conventional pharmacophore-based approaches to guide medicinal chemistry. More recently a similar approach was applied by Szabo et al [92]. They screened small fragments for allosteric activity versus the mGlu_2_ receptor and successfully identified a relatively low potency but high ligand efficiency benzotriazine fragment. Comparison with the pharmacophore in the Janssen reports suggested the optimal vectors for substitution to improve activity and more potent ligands were rapidly found.

Also based on the consistent ligand-based alignment hypothesis, scientists at Janssen demonstrated how computational QSAR and machine learning methods could be used to generate models to predict new potent substituents in a series of mGlu_2_ PAMs [93]. The newly identified compounds were highly potent in nearly all cases being better than 100 nM mGlu_2_ PAMs. The two different models had relatively low errors suggesting they were extremely valuable for compound activity prediction. The SVM derived model showed less tendency of overtraining compared to the 3D QSAR approach and gave better predictions over the duration of the project.

### 3.2. mGlu_3_ Receptor

The second group II mGlu receptor is mGlu_3_, and similar to mGlu_2_, it is also implicated in neuropsychiatric disorders such as schizophrenia [94]. Furthermore, the evolution of research around mGlu_3_ is closely intertwined with mGlu_2_ because the earliest small molecule synthetic orthosteric ligands designed for group II receptors suffered from limited selectivity [95]. Nowadays selective ligands exist and due to the single nucleotide polymorphisms in the GRM3 gene associated with schizophrenia, interest in the target is increasing.

Compared to mGlu_2_ there have been relatively few computational modeling studies of this receptor. Apart from the reports that have studied the whole mGlu family, specific focus on mGlu_3_ has involved work such as Yao et al. who built a homology model of the extra-cellular VFT and used it to study the binding mode of agonists [96]. Experimental mutagenesis revealed the important amino acids for [^3^H]DCG-IV binding and the model allowed visualization of the ligand in the binding site showing its important interactions.

The chloride ion has been shown to act as an agonist of group II and III mGlu receptors, and in particular it is an agonist of mGlu_3_ but not mGlu_2_ [97]. Mutagenesis and modeling showed the ion to bind in a cavity near the orthosteric binding site in the VFT domain. This small pocket is unavailable in mGlu_2_ receptors due to sequence differences. The phenomena of chloride ion activation were studied in more detail very recently in 2018 by Tora et al. [98]. They modeled the VFT domain and went on to show that Cl^−^ ions stabilize the glutamate-induced active state of the extracellular domain of the mGlu_3_ receptor and the large basal activity that had for many years been seen was due to chloride mediated positive allosteric modulation effect on low levels of residual glutamate concentrations. This is consistent with other recent reports of mGlu receptor activation being sensitive to residual levels of glutamate [99].

Finally, scientists at Lilly (Indianapolis, Indiana) have extensive experience in the field of orthosteric group II mGlu receptor ligands. Ever since the beginning of their work, computational methods have featured, such as with the design of conformational restrained glutamate analogs [100]. More up to date, they have released several reports of structure-based drug design efforts for group II agonists [101,102]. They used X-ray crystallography and docking and conformational modeling during the lead optimization of group II agonists to reach selective mGlu_3_ ligands [20].

## 4. Group III mGlu Receptors

### 4.1. mGlu_4_ Receptor

The mGlu_4_ receptor has emerged as an exciting therapeutic target for multiple indications such as Parkinson’s disease (PD) [103]. Agonists of group III mGlu receptors have shown robust efficacy in rodent models of PD including a highly selective mGlu_4_ receptor PAM, PHCCC. In addition to providing symptomatic relief for PD patients, several studies suggest that mGlu_4_ activation could provide a neuroprotective effect and slow disease progression.

In 2010 Slevam et al. identified new mGlu_4_ receptor agonists based on VS. Using their validated homology models of the mGlu_4_ binding pocket, they ran a VS campaign with the idea of finding new compounds that could reach other parts of the orthosteric pocket of mGlu_4_ to gain subtype selectivity. They examined if longer chain analogues of glutamate could still act as mGlu4 agonists, being able to reach less conserved regions outside the glutamate binding pocket [104]. Five new compounds were identified to activate mGlu_4,_ but only (R)-PCEP was chosen for further chemical optimization revealing a noticeable number of new group III mGlu receptor agonists.

In 2014 Rovira et al. studied ligands of mGlu_4_ receptor with the assistance of computational modeling and docking [105]. The modeling and docking studies identified two overlapping 7TM binding pockets as follows: a first site homologous to the pocket of natural agonists of class A GPCRs linked to allosteric agonism and a second one pointing toward a site topographically homologous to the Na+ binding pocket of class A GPCRs, occupied by PAMs exhibiting the strongest cooperativity. These results reveal that intrinsic efficacy and cooperativity of mGlu_4_ PAMs are correlated with their binding mode, and vice versa. Finally, as mentioned above, Dalton et al. studied a ligand with dual mGlu_4_ and mGlu_5_ functional activity using MD methods [67].

### 4.2. mGlu_7_ Receptor

Among the Group III mGlu receptors, mGlu_7_ is receiving increased attention. It is reported to be a potential target for numerous neurological and psychiatric disorders, such as: schizophrenia, epilepsy, anxiety and depression. It is differentiated from other members of group III due to its low affinity for glutamate binding. Amongst allosteric modulators, AMN082 was identified as the first selective allosteric-agonist of mGlu_7_, nevertheless the biology of mGlu_7_ is still only partially explored due to the difficulties to find potent selective tool compounds [11,106,107].

In 2017, scientists from Janssen reported a VS approach to find hits for mGlu_7_ positive allosteric modulation [108]. They used a method known as proteochemometrics that combines molecule and protein features into a single descriptor for each bioactivity point. The method is well suited to finding new hits for targets that are less explored, but where other protein family members have lots of associated screening data. The large numbers of bioactivity points available for molecules tested versus mGlu_1_, mGlu_2_ and mGlu_5_ for instance could be incorporated into a single model, and used to predict likelihood of activity versus mGlu_7_. The VS led to the identification of new mGlu_7_ allosteric modulators from several series. One of these was used as the starting point for a medicinal chemistry optimization program that was assisted with docking into a homology modeling of the mGlu_7_ receptor [109]. The compounds were rapidly optimized to nanomolar levels of activity validating the binding mode hypothesis and providing a promising series of mGlu_7_ PAMs.

### 4.3. mGlu_8_ Receptor

The mGlu_8_ receptor has been indicated in drug addiction [110] or anxiety [111], among a number of CNS disorders [112]. The structural opening and closing of the extracellular VFT domain have been discussed. Various studies that have included computational modeling have focused on elucidating the conformational changes of the extracellular domain of mGlu_8_ and its relation to receptor-specificity of orthosteric ligands targeting the receptor.

As early as 2002, Bessis et al. tried to elucidate the role of two antagonists in the closing of the VFT domain [113]. They modeled the mGlu_8_ receptor using the available mGlu_1_ crystal structure and then docked two antagonists (ACPT-II and MAP4) into the model. The antagonists were supposed to prevent the closing of the extracellular domain, hence hindering the activation of the receptor. They identified the key role of D309 and Y227 in the steric clash responsible for the inactivating action of ACPT-II and MAP4, further confirming the conformational changes in VFT needed for receptor activation.

Very recently, in 2018, three mGlu_8_ VFT domain crystal structures were solved. Schkeryantz et al. were the first to release the mGlu_8_ crystal in complex with l-glu and l-AP4 [114]. The binding mode of l-glu in this receptor differed from that previously described in mGlu_1_ crystals. In this work, they showed A154, A177 and E399 to be responsible for the differences observed with other mGlus. They also point to the key role played by a phosphate ion of l-AP4 in agonist potency and selectivity.

The last mGlu_8_ crystal structure was also released in 2018 by the same group., the mGlu_8_-(S)DCPG complex [112]. This structure differs from the previously solved (l-glu- and l-AP4 bound) by showing a larger opening angle between the two lobes of the domain. They also generated a homology model of mGlu_8_ using the mGlu_1_–l-glu crystal structure as template and docked (S)DCPG into it. Comparison between their crystallized complex, the previous structures, and the model uncovered the relevance of lobe opening angles, which may be considered when studying bulky agonists in future reports.

Feng et al. also built a homology model of the mGlu_8_ 7TM domain based on the mGlu_5_ crystal structure in their attempt to characterize the allosteric binding pocket of the non-crystallized mGlu receptors [66]. They docked NAM and PAM compounds to the model in order to identify residues involved in selectivity. Two different poses for AZ12216052 in mGlu_8_ were selected. Finally, they performed a 30 ns MD simulation of mGlu_8_ bound to AZ12216052 PAM to validate the stability of the complex and the ligand-receptor interactions implicated. Results showed a binding pose of this ligand that involved S663 and S829 forming key H-bonds with the PAM. With the recent release of extracellular domain crystal structures of mGlu_8_ bound to three different agonists, new opportunities to better understand the orthosteric pocket of the receptor arise. However, mGlu_8_ allosteric regulation still represents a challenge and there has been relatively little computational work in the area.

## 5. Conclusions

The mGlu_1_ and mGlu_5_ receptors have been studied in a relatively similar manner with computational approaches. Before their crystal structures were known most studies relied on ligand-based approaches to better understand these receptors focusing on virtual screening and pharmacophore building approaches. Homology models based on class A GPCR templates available at the time were built to map the mutagenesis data. With the solving of the 7TM domain structures of mGlu_1_ and mGlu_5_ in 2014, the data collected up to then could be validated. Moreover, the confirmation of the allosteric pocket location in the 7TM domain opened new horizons for structure-based drug design studies targeting mGlu receptors. Also, the release of four more NAM bound crystallographic 7TM structures of mGlu_5_ provided further insight in the binding mode of different scaffolds to this receptor. However, the activation mechanism and PAM SARs still represent a challenge since no available structure of an active state of the receptor had been released but modeling work on molecular switches has suggested new insights. The new dimeric full length structures of mGlu_5_ open great opportunities for better understanding of these aspects.

In group II, the mGlu_2_ receptor has been widely studied. An array of ligand and structure-based approaches have been used. The use of ligand-based approaches has been shown to have a significant impact on medicinal chemistry by identifying new hits from VS and scaffolds for lead series. Meanwhile, the experimental mutagenesis was supported with molecular modeling to confirm the allosteric binding site. A series of detailed MD studies followed that have demonstrated a hypothesis for the origins of PAM versus NAM activity. The PAMs generate increased movement amongst amino acid sidechains of the transmission switch, whilst the NAMs generally stabilize amino acids in the inactive-like state, showing less movement, consistent with MD simulations and analyses on mGlu_5_. Such results can offer great promise if they can be extrapolated to the design of new compounds.

Group III mGlu receptors remain the least studied of the family, yet they offer great therapeutic opportunity. Recently, VS studies led to identifying promising compounds targeting mGlu_4_, mGlu_7_ and mGlu_8_ receptors. To better understand the selectivity of these compounds for each receptor, modelling and docking approaches were applied. Nevertheless, this group of mGlu receptors still needs to be further characterized.

MD has been used in several studies to understand details of functional activity [72,82,84,85]. As the new structure of the full length mGlu_5_ receptor has revealed the large-scale motions that accompany activation, it will be fascinating to see in later studies how more local conformational changes affect functional activity. The binding site of class A orthosteric ligands overlaps with class C allosteric modulators. However, the allosteric ligands are not able to activate the receptor alone. The microswitch hypothesis proposes that certain groupings of amino acids which are spatially co-located can translate ligand induced effects into knock-on movements that stabilize active states, promoting more activation of the receptor population and that this can be relevant for mGlu receptor allosteric modulation [72,82]. Therefore, will the hypothesis of translating features for class A activation to class C allosteric ligands hold true? In terms of future computational approaches, there has been increased interest in both machine learning and physics based computational modeling. The former is well suited to GPCRs as it relies upon using available data to make future predictions. Such methods have already shown promise for mGlu receptors [60,109]. On the other hand, there are no reports on the application of physics-based methods such as free energy perturbation for mGlu receptors. It is expected that the future will provide significant opportunities for more quantitative mGlu receptor drug design, building on early studies demonstrating the feasibility of these approaches for GPCRs in general [115].

## Figures and Tables

**Table 1:** Summary of crystal structures for the 7TM domain of mGlu receptors.

Receptor	PDB ID	Method	Resolution	Description	Reference
mGlu_1_	4OR2	X-ray diffraction	2.8 Å	Crystal structure of 7TM domain of human mGlu_1_ bound to FITM (NAM). Soluble cytochrome b562 present for stabilization.	[17]
mGlu_5_	4OO9	X-ray diffraction	2.6 Å	Structure of the human 7TM domain of mGlu_5_ bound to mavoglurant (NAM). Lysozyme used for stabilization.	[16]
	5CGC	X-ray diffraction	3.1 Å	Structure of human 7TM domain of mGlu_5_ bound to 3-chloro-4-fluoro-5-[6-(1*H*-pyrazol-1-yl)pyrimidin-4-yl]benzonitrile (NAM). Endolysin present in the structure used for stabilization.	[18]
	5CGD	X-ray diffraction	2.6 Å	Structure of human 7TM of mGlu_5_ bound to HTL14242 (NAM). Endolysin present in the structure used for stabilization.	[18]
	6FFH	X-ray diffraction	2.7 Å	Crystal structure of 7TM domain of human mGlu_5_ bound to fenobam (NAM). Endolysin present in the structure for stabilization.	[19]
	6FFI	X-ray diffraction	2.2 Å	Crystal structure of 7TM domain of human mGlu_5_ bound to M-MPEP (NAM). Endolysin present for stabilization.	[19]
